# Magnetic compression anastomosis for non-anastomotic stenosis of the proximal jejunum after total gastrectomy with Roux-en-Y reconstruction: a case report

**DOI:** 10.1186/s40792-020-00932-8

**Published:** 2020-07-09

**Authors:** Teppei Kamada, Hironori Ohdaira, Hideyuki Takeuchi, Junji Takahashi, Rui Marukuchi, Norihiko Suzuki, Satoshi Narihiro, Sojun Hoshimoto, Masashi Yoshida, Eigoro Yamanouchi, Yutaka Suzuki

**Affiliations:** 1 Department of Surgery, International University of Health and Welfare Hospital, 537-3 Iguchi, Nasushiobara, Tochigi, 329-2763 Japan; 2 Department of Radiology, International University of Health and Welfare Hospital, 537-3 Iguchi, Nasushiobara, Tochigi, 329-2763 Japan

**Keywords:** Magnetic compression anastomosis, Non-anastomotic stenosis, Balloon dilation

## Abstract

**Background:**

Postoperative non-anastomotic stenosis of the proximal jejunum after total gastrectomy with Roux-en-Y reconstruction is a rare complication. If endoscopic balloon dilation proves ineffective, patients need re-operation under general anesthesia and experience a high rate of postoperative complications. Magnetic compression anastomosis is a nonsurgical procedure that can create an anastomosis similar to that obtained through surgery. We report a case in which magnetic compression anastomosis was successfully performed to avoid re-operation for non-anastomotic stenosis of the proximal jejunum after total gastrectomy with Roux-en-Y reconstruction.

**Case presentation:**

A 70-year-old woman was admitted to our hospital for treatment of non-anastomotic stenosis of the proximal jejunum. Open total gastrectomy and Roux-en-Y reconstruction were performed 2 years previously for advanced gastric cancer at another hospital. She complained of anorexia and obstructed passage of food. No recurrence of gastric cancer was identified. Esophagogastroduodenoscopy showed circumferential membranous stenosis of the jejunum 3 cm distal to the esophago-jejunal anastomosis. Endoscopic balloon dilation was performed three times, but proved ineffective. Magnetic compression anastomosis was planned because the stenosis existed near the esophago-jejunal anastomosis and re-operation was a highly invasive procedure requiring intrathoracic anastomosis. Endoscopic balloon dilation preceded placement of the parent magnet on the anal side of the stenosis. Confirming the improvement of stenosis, the parent magnet was placed on the anal side of the stenosis during esophagogastroduodenoscopy. The parent magnet attached to nylon thread was fixed to the cheek to prevent magnet migration. A week after placing the parent magnet, restenosis was confirmed and the daughter magnet was placed via nylon thread on the oral side of the stenosis. The two magnets were adsorbed in the end-to-end direction across the stenosis. Magnets adsorbed in the end-to-end direction moved to the anal side 11 days after placement. Wide anastomosis was confirmed by esophagogastroduodenoscopy. Endoscopic balloon dilation was regularly performed to prevent restenosis after magnetic compression anastomosis. No complications were observed postoperatively. The patient was able to eat normally and successfully reintegrated into society.

**Conclusions:**

Magnetic compression anastomosis could be a feasible procedure to avoid surgery for non-anastomotic stenosis of the proximal jejunum after gastrectomy with Roux-en-Y reconstruction.

## Background

Postoperative non-anastomotic stenosis of the proximal jejunum after total gastrectomy with Roux-en-Y reconstruction (R-Y reconstruction) is a rare complication. Non-anastomotic stenosis can be caused by problems, such as intra-abdominal adhesions, kinking, internal hernia of esophageal hiatus, or local recurrence of tumors [1, 2].

Most patients diagnosed with non-anastomotic stenosis without improvement of conservative therapy need endoscopic balloon dilation (EBD), which is an effective procedure. However, some patients need re-operation under general anesthesia for both diagnosis and treatment or endoscopic and/or fluoroscopic self-expandable metallic stent (SEMS) placement when their situation does not improve. Such cases usually show a high rate of postoperative complications [3, 4].

Magnetic compression anastomosis (MCA) has recently been reported as a minimally invasive treatment to avoid surgery under general anesthesia. MCA is a novel interventional method that creates an anastomosis using two rare earth magnets in the targeted segments of the gastrointestinal tract. MCA is mainly applied in the gastrointestinal and biliary tracts and is a nonsurgical procedure that can create an anastomosis similar to that obtained surgically. MCA can be applied even for patients with ascites caused by peritoneal dissemination or patients who are unable to undergo general anesthesia [5].

Some researchers have reported MCA as a feasible method for benign stricture of duct-to-duct biliary anastomosis after living-donor liver transplantation [6]. We report a case in which MCA was successfully performed to avoid re-operation and ingestion is enabled for non-anastomotic stenosis of the proximal jejunum after total gastrectomy with R-Y reconstruction for gastric cancer.

## Case presentation

**Fig. 1:**
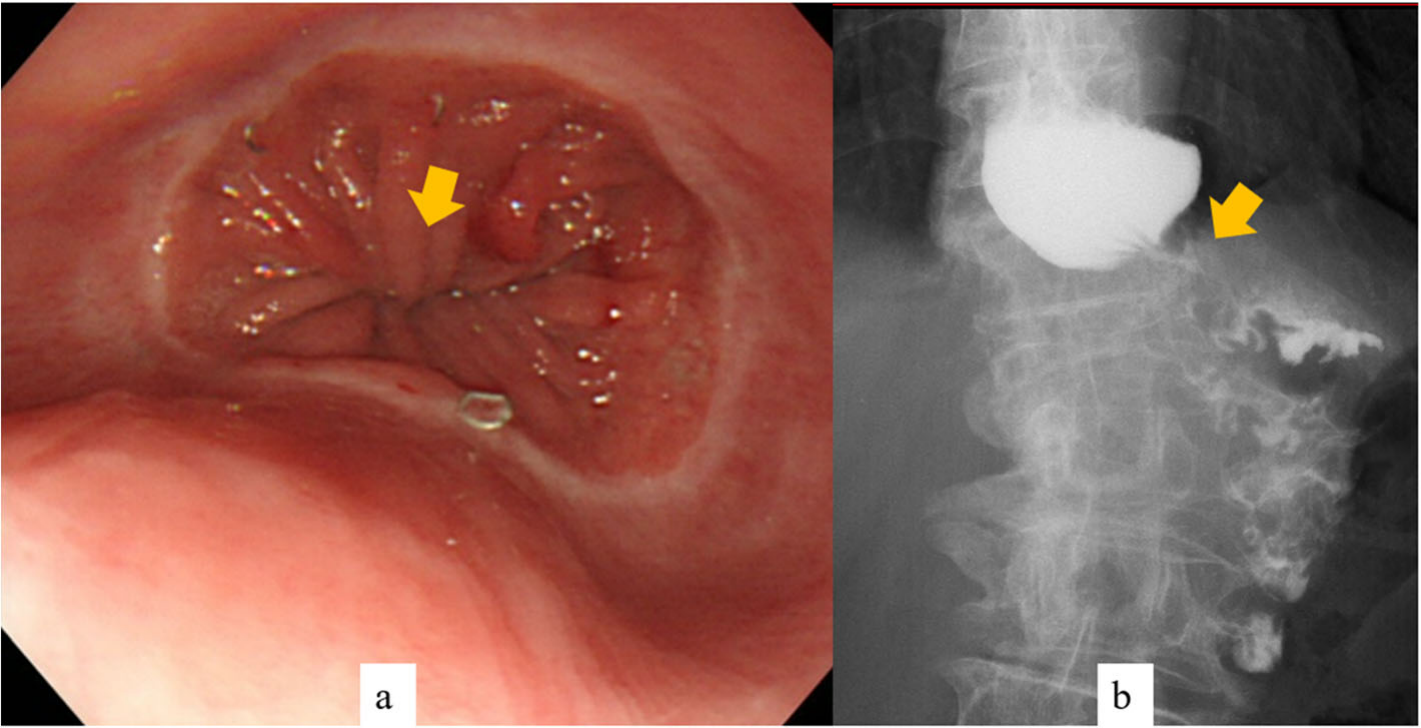
Circumferential membranous stenosis of the jejunum 3 cm distal to the esophago-jejunal anastomosis. **a** Stenosis observed by esophagogastroduodenoscopy (arrow). **b** Stenosis observed by upper gastrointestinal fluoroscopy (arrow)

A 70-year-old woman was admitted to our hospital to treat non-anastomotic stenosis of the proximal jejunum. Open total gastrectomy and R-Y reconstruction had been performed at another hospital 2 years previously for advanced gastric cancer (pT3N2M0, stage IIIA). No complications were observed during the postoperative course, and no recurrence of cancer was apparent for 2 years. She complained of anorexia and obstruction to the passage of food. Recurrence of gastric cancer was ruled out based on blood test results and various image findings. Esophagogastroduodenoscopy showed circumferential membranous stenosis of the jejunum 3 cm distal to the esophago-jejunal anastomosis (Fig. 1a). Upper gastrointestinal fluoroscopy showed that the length of the stenotic region was almost 0.5 cm (Fig. 1b).

EBD was performed three times. Symptoms improved after EBD in the early days, but restenosis occurred within 1 week and proved difficult to treat. MCA was planned because the stenosis existed near the esophago-jejunal anastomosis and re-operation is a highly invasive procedure requiring intrathoracic anastomosis. The protocol used for MCA was approved by the Ethics Committee for Biomedical Research of the International University of Health and Welfare Hospital, and the patient provided informed consent (approval no. 13-B-90).

**Fig. 2:**
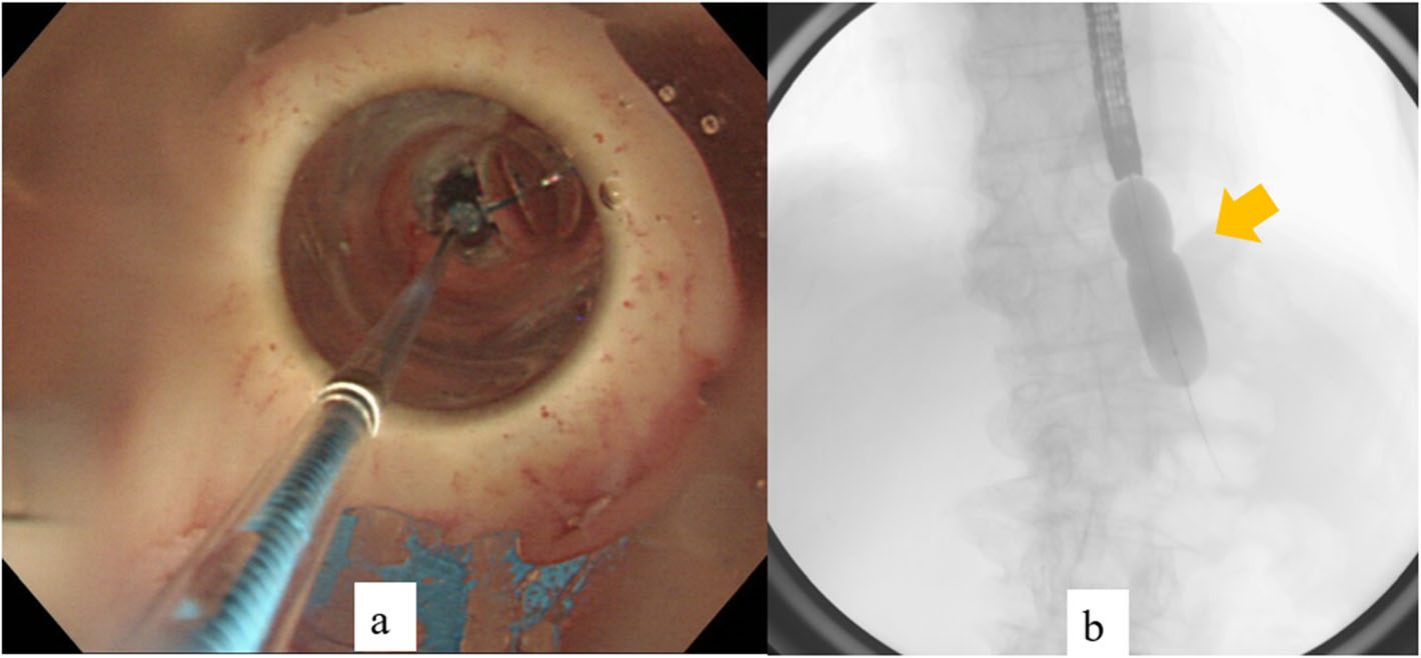
Endoscopic balloon dilation. **a** Esophagogastroduodenoscopy imaging. **b** Radiograph showing the “waist” caused by stenosis

**Fig. 3:**
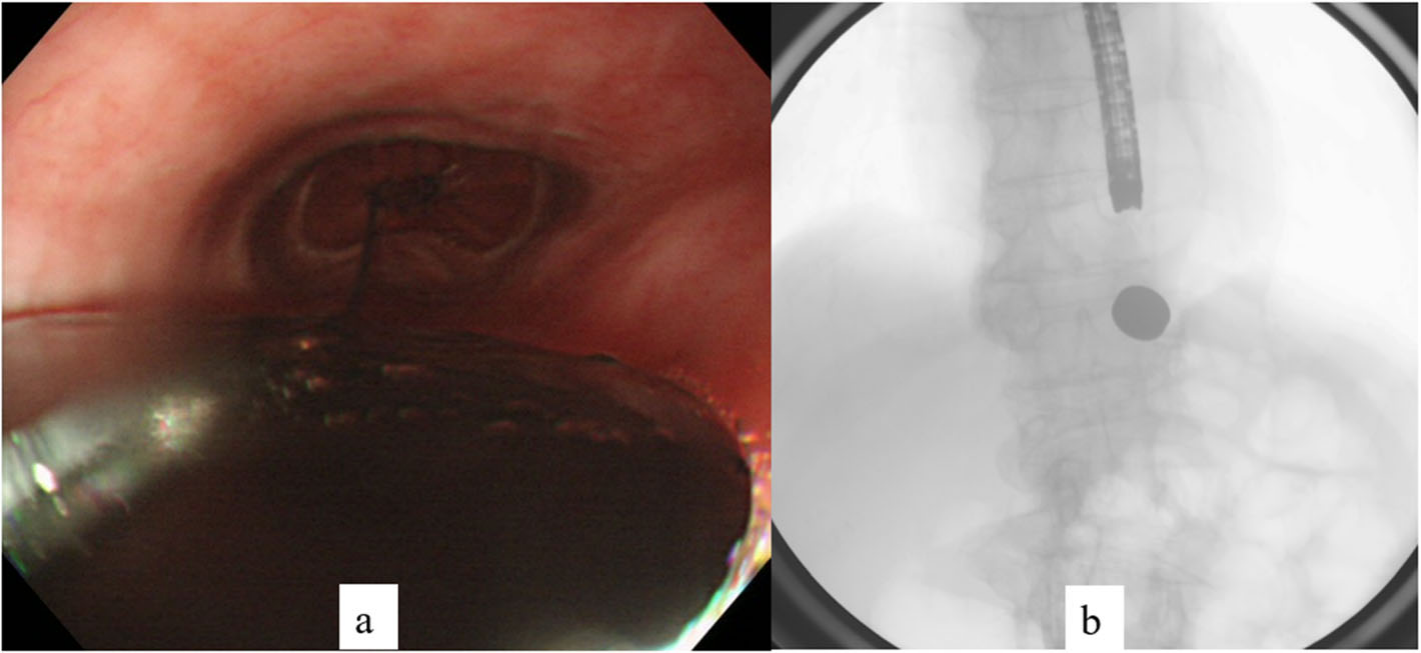
Magnetic compression anastomosis (placing the parent magnet on the anal side of the stenosis). **a** Esophagogastroduodenoscopy. **b** Radiograph

**Fig. 4:**
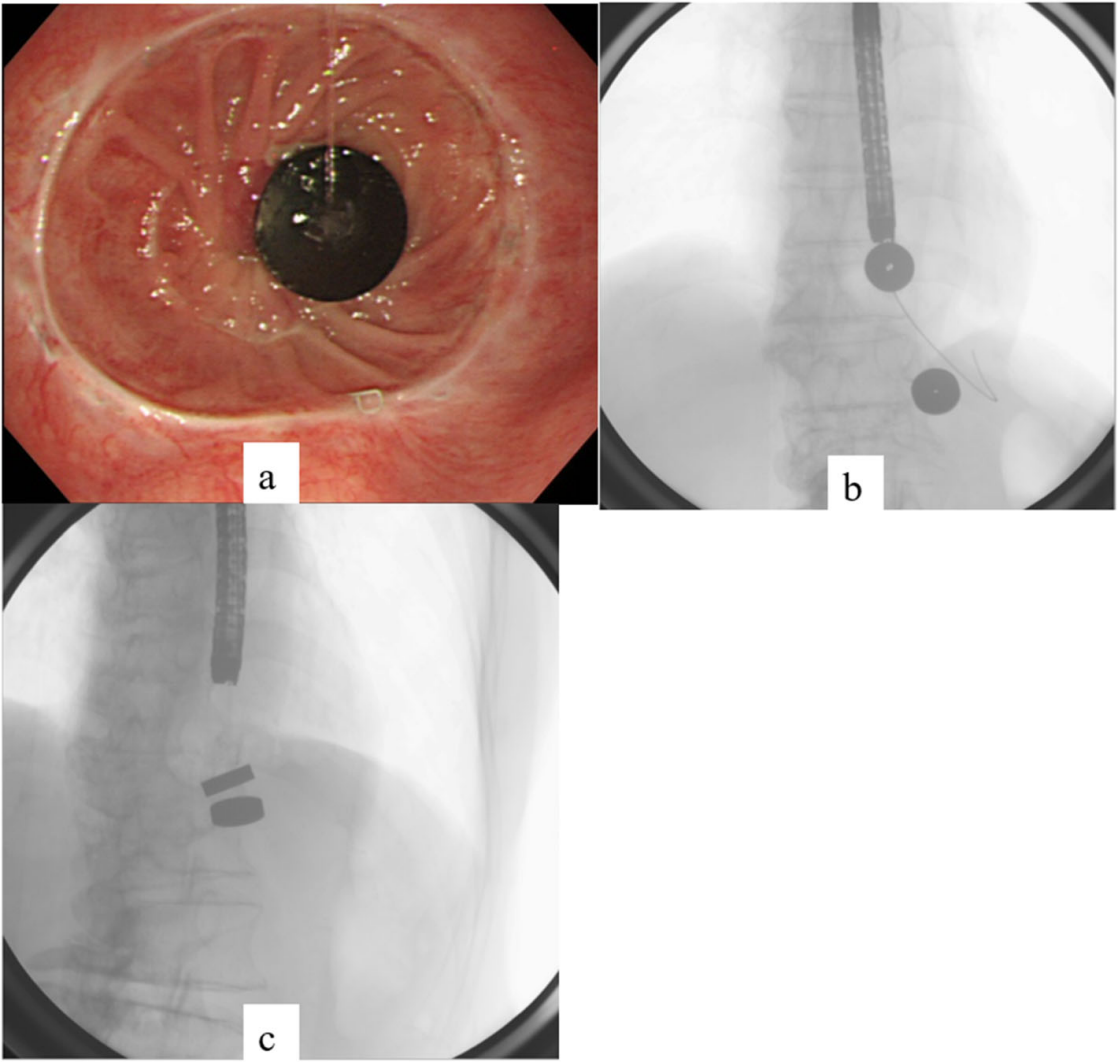
Magnetic compression anastomosis (placing the daughter magnet on the oral side of the stenosis). **a** Esophagogastroduodenoscopy. **b** Radiograph. **c** Radiograph showing adsorption of the two magnets in the side-to-side direction

**Fig. 5:**
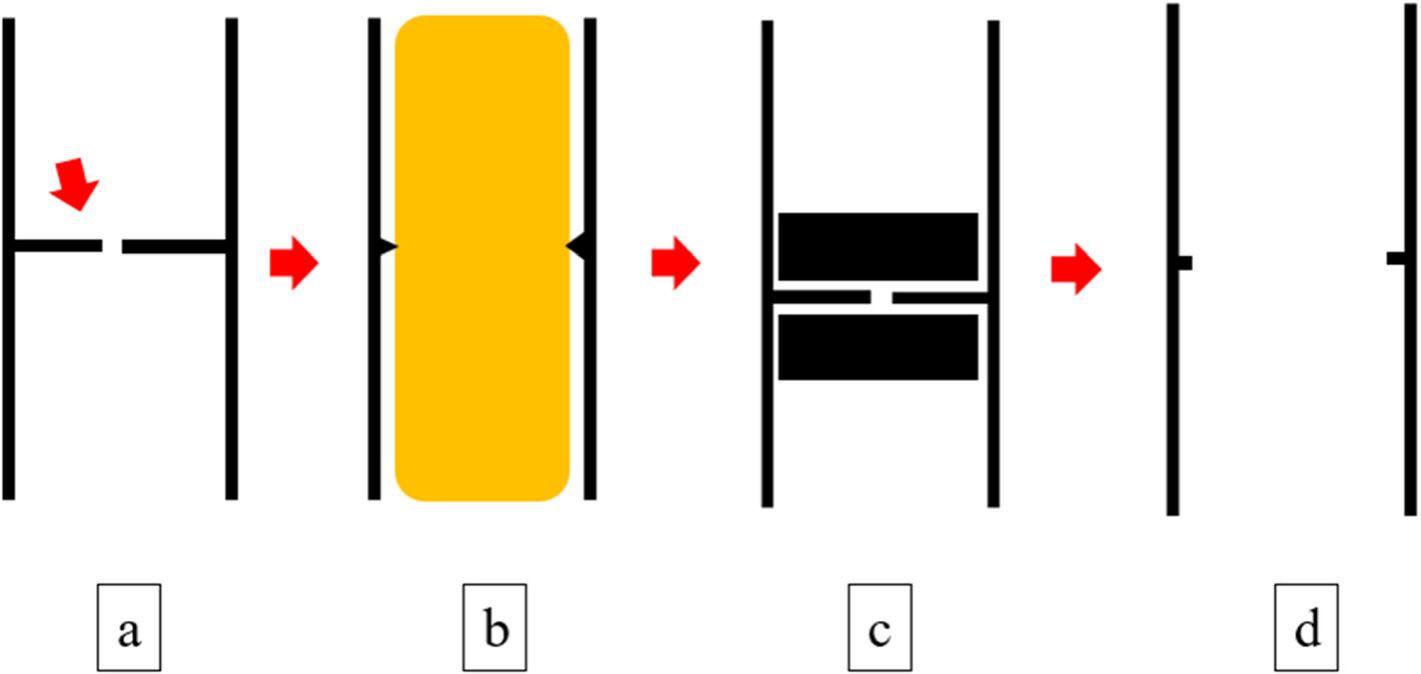
Schema of current treatment. **a** Pre-treatment imaging (arrow, stenosis). **b** Balloon dilation. **c** Adsorption of two magnets across the stenosis. **d** After-treatment imaging

**Fig. 6:**
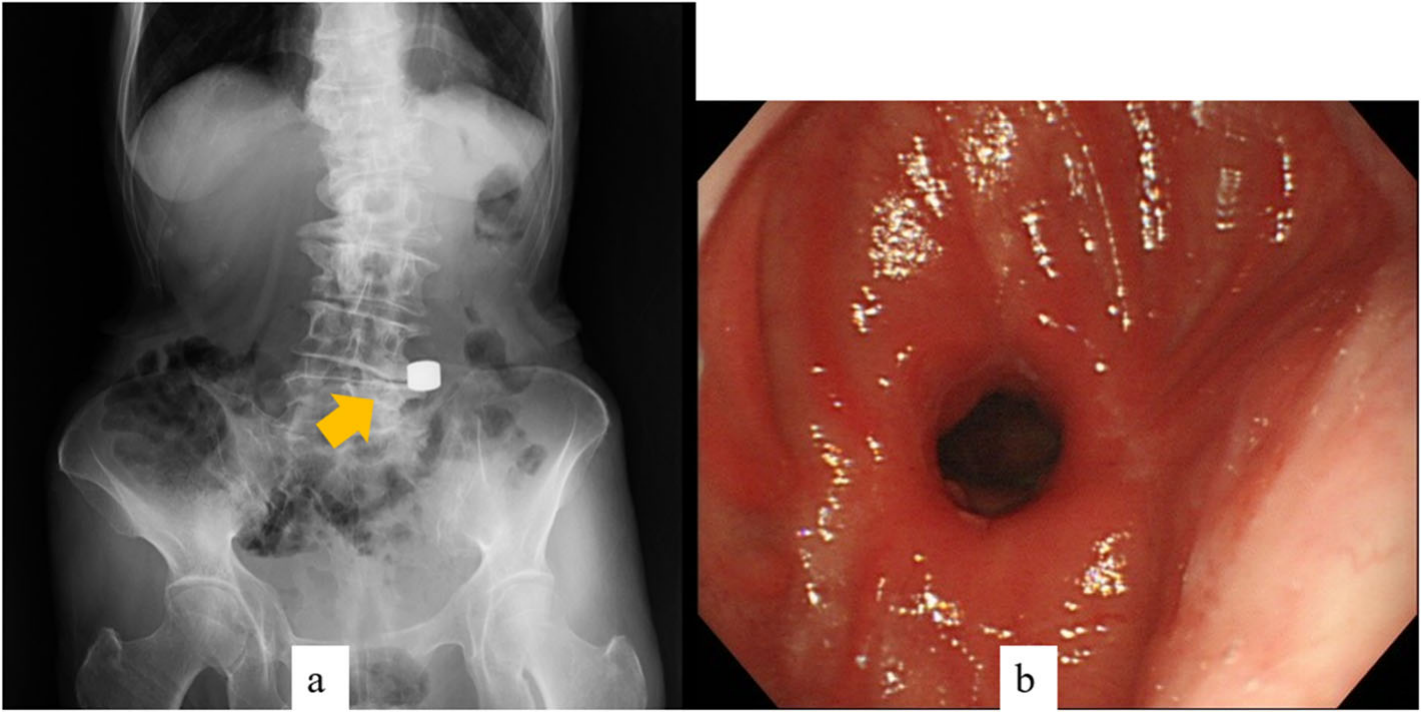
Imaging of the created anastomosis. **a** Plain X-ray imaging: two magnets have moved to anal side (arrow). **b** Anastomosis observed by esophagogastroduodenoscopy after removal of the magnets

Both the parent magnet (diameter, 17.5 mm; thickness, 5 mm) and daughter magnet (diameter, 17.5 mm; thickness, 5 mm) were cylinders made of samarium-cobalt (Magna Corporation, Shinjuku, Tokyo). EBD was performed to place the parent magnet on the anal side of the stenosis. A wire-guided balloon dilation catheter (CRE balloon catheter; Boston Scientific, Natick, MA) was inserted and EBD (7 atm-17 mm, 3 min) was performed (Fig. 2). Confirming the improvement of stenosis, the parent magnet was placed on the anal side of the stenosis using esophagogastroduodenoscopy (Fig. 3). The parent magnet attached to nylon thread was fixed to the cheek to prevent migration. A week after placing the parent magnet, restenosis was confirmed and the daughter magnet was placed via nylon thread on the oral side of the stenosis using esophagogastroduodenoscopy (Fig. 4a, b). The two magnets were adsorbed in the end-to-end direction across the stenosis (Figs. 4c and 5). Magnet position was confirmed on X-ray each day. The magnets adsorbed in the end-to-end direction moved to the anal side by 11 days after treatment and were passed from the anus 14 days after treatment (Fig. 6a). Wide anastomosis was confirmed by esophagogastroduodenoscopy (Fig. 6b). No complications were observed in the postoperative course of this patient. Oral intake was resumed on postoperative day 7, and the patient was discharged on postoperative day 21.

EBD was regularly performed to prevent restenosis on an outpatient basis (first, every 1 week, 2 weeks, 1 month, 4 months). Eighteen months has passed after treatment, and no restenosis of MCA anastomosis has occurred. We judged that no further EBD was required. The patient has resumed eating normal food and has reintegrated into society.

## Conclusions

Postoperative non-anastomotic stenosis of the proximal jejunum after total gastrectomy with R-Y reconstruction is a rare complication. Early non-anastomotic stenosis within the first 30 postoperative days after reconstructive surgery can be caused by technical problems such as kinking, narrowing or acute angulation of the anastomosis, or internal herniation of the esophageal hiatus, while late non-anastomotic stenosis (occurring after > 1 year) can occur secondary to intra-abdominal adhesions, kinking, osmotic or ischemic changes of the proximal jejunum, or recurrence of tumors.

Recurrence of gastric cancer was ruled out based on results of blood tests and various image findings.

EBD was performed several times, and symptoms improved after EBD in the early days, but restenosis occurred within a week and proved difficult to treat.

Recently, endoscopic or fluoroscopic balloon dilation has been shown to offer a feasible procedure to avoid re-operation for non-anastomotic stenosis of the proximal jejunum. Tsauo et al. suggested that 82.1% of patients achieved clinical success after a single fluoroscopic balloon dilation, but 26.5% of patients experienced recurrence within 1 year after fluoroscopic balloon dilation [3]. In another study, recurrence of symptoms of non-anastomotic stricture of the proximal jejunum occurred after 64.7% (11/17) of procedures [4].

Endoscopic and/or fluoroscopic SEMS placement is the first-line palliative option for esophageal and gastrointestinal stenosis, alternative to balloon dilation. Bakheet et al. suggested that fluoroscopic SEMS placement may be effective and safe for treating postoperative non-anastomotic strictures, but stent malfunction and recurrence are major drawbacks [4]. In their study, stent malfunction occurred after 58.8% (10/17) of procedures, including six occurrences of stent migration and four of benign tissue hyperplasia. Furthermore, complications of SEMS placement include perforation or obstruction of the digestive tract [7, 8], and few studies have examined long-term outcomes of SEMS placement. This is why MCA was introduced for benign stenosis in patients who are unable to undergo surgery in our institution, if endoscopic balloon dilation proves ineffective.

MCA is a safe and unique technique for the reconstruction of complicating entericoenteric, biliobiliary, or bilioenteric anastomoses without surgical intervention.

Yamanouchi et al. [5] reported the first use of MCA in the 1990s and suggested a high success rate (almost 100%) and low complication rate (3.2%), including anastomotic leakage or other organ injury in 62 cases [9]. MCA has been performed to treat complications such as stenosis of the bile duct after intraoperative bile duct injury for patients who are unable to undergo general anesthesia [10–13].

However, few reports have described the use of MCA in the gastrointestinal area. Kawabata et al. reported two cases in which MCA was applied to address gastrointestinal stenosis. One case involved anastomotic obstruction after subtotal gastrectomy, and the other involved endoscopic gastrojejunostomy for superior mesenteric artery syndrome. In those reports, the magnets measured 15 × 3 mm, and the duration until creation of complete anastomosis was 10 days each, similar to our case [14, 15].

The essential point of MCA in the current case was that EBD was performed first to place the 17.5-mm parent magnet on the anal side of the stenosis.

Confirming that restenosis had occurred, the daughter magnet was placed via nylon thread on the oral side of the membranous circumferential stenosis using esophagogastroduodenoscopy, resulting in shaving off the membrane of the scar tissue.

If the daughter magnet is adsorbed to the parent magnet just after EBD, MCA is not effective, because the adsorption area is so small that the residual membrane of the scar tissue is present. That is why the daughter magnet was placed 7 days after EBD with restenosis. During this period, we prevented the parent magnet from migrating using nylon thread attached to the daughter magnet, fixed to the cheek. EBD should be performed regularly to prevent restenosis after MCA. The rate of restenosis after MCA is reportedly about 20% [9]. In the current case, we gradually lengthened the interval of EBD and finally judged that no further treatment was needed.

EBD alone is not effective, but MCA + EBD appears effective. This fact suggested that restenosis can be caused by the membrane of the scar tissue. Cheng et al. elucidated the mechanism of restenosis following balloon dilation of benign esophageal stenosis [16]. The esophageal morphology was altered by balloon catheter dilation. The esophageal mucosa exhibited not only chemical burn lesions, but also lesions caused by mechanical damage. The thickened muscle layer of the esophagus was torn or broken, causing the areas of the mucosal and muscle layers of the esophagus to increase significantly in the experimental group. Up to a certain time, these new scar tissues of mucosa would further contract and cicatrize. As a result, the duct lumen was further reduced and lacked elasticity [16]. Restenosis of the jejunum as in the current case is considered to involve the same mechanisms. Shaving off only the membrane of the scar tissue by MCA creates a new mucosa without creating further mucosal scarring.

MCA has been reported to show few complications, but anastomotic leakage, restenosis, injury to other organs, and deviation and aberration of the magnets are sometimes reported [9]. Furthermore, MCA is not applicable for some cases, such as where the parent magnet cannot be placed on the anal side of the severe stenosis or malignant stenosis of the digestive tract is identified.

However, our experience suggests that MCA could offer an alternative procedure to avoid surgery for non-anastomotic stenosis of the proximal jejunum after total gastrectomy with R-Y reconstruction.

In conclusion, MCA could provide a feasible procedure to avoid surgery for non-anastomotic stenosis of the proximal jejunum after gastrectomy with R-Y reconstruction.

## Data Availability

Data sharing is not applicable to this article, as no datasets were generated or analyzed during the current study.

## Abbreviations

R-Y reconstruction: Roux-en-Y reconstruction
EBD: Endoscopic balloon dilation
SEMS: Self-expandable metallic stent
MCA: Magnetic compression anastomosis
